# High Performance GPU-Based Fourier Volume Rendering

**DOI:** 10.1155/2015/590727

**Published:** 2015-02-19

**Authors:** Marwan Abdellah, Ayman Eldeib, Amr Sharawi

**Affiliations:** Biomedical Engineering Department, Cairo University, Giza 12613, Egypt

## Abstract

Fourier volume rendering (FVR) is a significant visualization technique that has been used widely in digital radiography. As a result of its *𝒪*(*N* ^2^log⁡*N*) time complexity, it provides a faster alternative to spatial domain volume rendering algorithms that are *𝒪*(*N* ^3^) computationally complex. Relying on the *Fourier projection-slice theorem*, this technique operates on the spectral representation of a 3D volume instead of processing its spatial representation to generate attenuation-only projections that look like *X-ray radiographs*. Due to the rapid evolution of its underlying architecture, the graphics processing unit (GPU) became an attractive competent platform that can deliver giant computational raw power compared to the central processing unit (CPU) on a per-dollar-basis. The introduction of the compute unified device architecture (CUDA) technology enables embarrassingly-parallel algorithms to run efficiently on CUDA-capable GPU architectures. In this work, a high performance GPU-accelerated implementation of the FVR pipeline on CUDA-enabled GPUs is presented. This proposed implementation can achieve a speed-up of 117x compared to a single-threaded hybrid implementation that uses the CPU and GPU together by taking advantage of executing the rendering pipeline entirely on recent GPU architectures.

## 1. Introduction

Volume visualization is an essential tool for exploring and analysing the anatomy of complex structures and phenomena. It has been extensively used in various scientific and engineering arenas such as medical imaging, geoscience, microscopy, mechanical engineering, and others [1–3]. Several volume visualization techniques have been developed and extensively investigated. The main two categories that have gained broad acceptance by scientific communities were* volume* and* surface rendering*. Each technique has its specific area of application that is associated with its advantages, but in contrast, it also has its disadvantages that give the opportunity for other techniques to survive [4–6].

Volume rendering has shown great significance on the visual interpretation of large amount of 3D scalar and vector data generated by multidimensional sensors, acquisition devices, and supercomputer simulations. It concentrates on visualizing the desired internal features of volumetric objects and their bounding surfaces at the same time. In literature, several volume rendering algorithms have been presented either to improve the rendering speed of large datasets or to enhance the rendering quality of their reconstructed images. By and large, the rendering speed and quality are* traded-off* and no single complete algorithm that can deliver the optimum quality associated with maximum interactivity exists [7].

A radical* domain-based* categorization of volume rendering algorithms classifies them into* spatial-domain* and* other-domain-based* techniques such as frequency domain, compression domain or the wavelet domain. The rendering pipeline of spatial domain techniques runs entirely in this domain. Domain-based methods operate by switching part of their computations to a different domain either to reduce the complexity of the rendering algorithm or to improve the performance of some operations that take considerable amount of time in the spatial domain. For a volume of size *N* ^3^, complexity of spatial-domain algorithms is of order *𝒪*(*N* ^3^), since all the voxels composing this volume must be visited at least once to render a correct image. Although some algorithms use additional optimization techniques to reduce the number of traversed samples, this optimization is in general data dependent and subject to the size of the datasets. This time-complexity limits the usage of spatial domain rendering algorithms for interactive environments in some applications. In such cases,* frequency-domain-based* techniques can be used alternatively.

Frequency-domain volume rendering (FDVR) uses a 3D spectral representation of the volume to compute an image that looks like X-ray radiograph in *𝒪*(*N* ^2^log⁡⁡*N*) time relying on the* projection-slice theorem*. It works by transforming the spatial volume into frequency domain. Then it reconstructs 2D projection at any viewing angle by resampling an extracted projection-slice along a perpendicular plane to the viewing direction followed by backtransforming this resampled slice to the spatial domain [8, 9].

Obtaining the spectral representation of the volume is the most computationally intensive step in the algorithm due to its *𝒪*(*N* ^3^log⁡⁡*N*) complexity. Nevertheless, this rendering algorithm is extremely efficient because this step is executed once in a preprocessing stage. The rendering loop of this pipeline takes much less computing time.

FDVR is a generic technique that can work with any frequency transform to switch between the spatial and frequency domains. Our rendering pipeline will be based on Fourier transform and so, the rendering method can be called Fourier volume rendering.

The rest of the paper is organized as follows.  Section 2 summarizes the related work in the literature. In Section 3, the rendering algorithm is demystified and our implementation strategy is presented.  Section 4 elaborates the results gained by mapping the entire rendering pipeline to run on the GPU and Section 5 concludes the paper.

## 2. Fourier Volume Rendering Literature

Although the technique was introduced around 20 years ago, the literature behind FDVR in general and FVR in particular is quite scarce. In this section, we will try to summarize the most notable contributions that have been presented since that time in a nutshell.

In 1992, Dunne et al. introduced the fundamental idea behind frequency domain volume rendering and its advantages [8]. Malzbender systematically extended the projection-slice theorem to 3D to be a basis for volume rendering [9]. Based on FFT, he presented an implementation of the basic FVR pipeline and briefly discussed some considerations of resampling the frequency domain and their significant effects on the reconstruction quality of the generated projection image. He proposed to use a Hamming-windowed *sinc* reconstruction filter to reduce the aliasing accompanied with resampling the frequency spectrum. For the same mission, Grosso and Ertl proposed alternatively biorthognal wavelet reconstruction filters [10]. As a drawback, the basic FVR integral does not provide a direct way of modelling emission and scattering in the participating medium. This limitation was a fundamental disadvantage associated with frequency domain rendering that is reflected as lack of occlusion in the resulting reconstructions [11]. Levoy has partially restored the lost visual cues in the basic pipeline by applying several shading models that are linear combinations of Fourier projections [12]. His extension included depth cueing, directional shading, and Lambertian reflection with spherical illumination. The result of Levoy's work did not exhibit real occlusion, yet it provided acceptable depth and shape cues that can simulate the existence of the missing occlusion. One significant disadvantage that limited the usage of his implementation was the demand of several copies of the volume, which consequently imposes high memory requirements for initial preprocessing. In cooperation with Totsuka, the same results have been obtained after considering alternative frequency domain methods by processing the frequency response of the volume data, which dramatically reduced the memory required before [13].

Until that time, FVR did not gain that broad acceptance by the scientific visualization and medical communities due to the lack of illumination models in the Fourier domain. In 2002, Entezari et al. enhanced the projections quality by incorporating various illumination models into the FVR pipeline [14]. The first model was based on Gamma-corrected hemispherical shading that was proposed by Scoggins et al. [15] and the second one adopted spherical harmonic functions for approximating cubic illumination shading. FVR suffered from reduced reconstruction quality that limited its usefulness in particular medical applications. In [16], a solution for enhancing FVR using contour extraction was proposed. It provided a flexible method for extracting material boundaries on surfaces in the Fourier space. Additionally, it included enhancement of several features for revealing important spatial relationships between interior and exterior structures making it an attractive tool for improved X-ray-like investigations for a given dataset. Cheng and Ching [17] also presented various methods for designing FVR transfer function based on Bezier curves and B-splines. Jansen et al. [18] partially accelerated the rendering pipeline on the GPU by mapping the Split-Stream-FFT on the GPU. As a major tool in digital radiography, Ntasis et al. [19] provided a web-based Fourier volume renderer for real-time preview of digital reconstructed radiographs (DRRs). Their implementation examined carefully the resampling issues of the frequency domain to generate remote high quality DRRs. Extending the technique beyond operating on volume data with regular grids, FVR has been adapted by Corrigan et al. [20, 21] to directly deal with meshless data. A high-level MATLAB-based FVR framework was presented in [22]. This framework abstracts the complex implementation details of the rendering pipeline to allow imaging researchers to develop image enhancement techniques without having prior knowledge of the OpenGL pipeline. Viola et al. have implemented a FVR heterogeneous rendering pipeline that uses the GPU to execute the rendering stage relying on fragment shaders [23]. However, this approach is limited if the current capabilities of unified computing GPU architectures are considered.

Practical implementation of FVR is complicated by two main factors which arise when the projection-slice theorem is applied to discrete sampled data [8]. First of all, conventional FFT algorithms yield frequency-domain output data which is not ideally structured for resampling. To mitigate this effect, it is necessary to add high performance multidimensional fft-shift stages to the rendering pipeline to rearrange the data. Moreover, sampling the frequency domain is equivalent to the replication of the signal in the spatial domain that ultimately accounts for the appearance of ghosting artifacts in the reconstructed images. This issue is resolved by zero-padding the volume in the spatial domain and using high-order interpolation filters in the frequency domain. Our CUDA-based implementation addresses all of these issues.

## 3. Algorithm and Implementation

The plain FVR algorithm is briefly illustrated to simplify the explanation of the contextual classification of the rendering pipeline. Based on this classification, an efficient strategy has been considered to map this pipeline to run entirely on the GPU.

### 3.1. Algorithm

**Figure 1:**
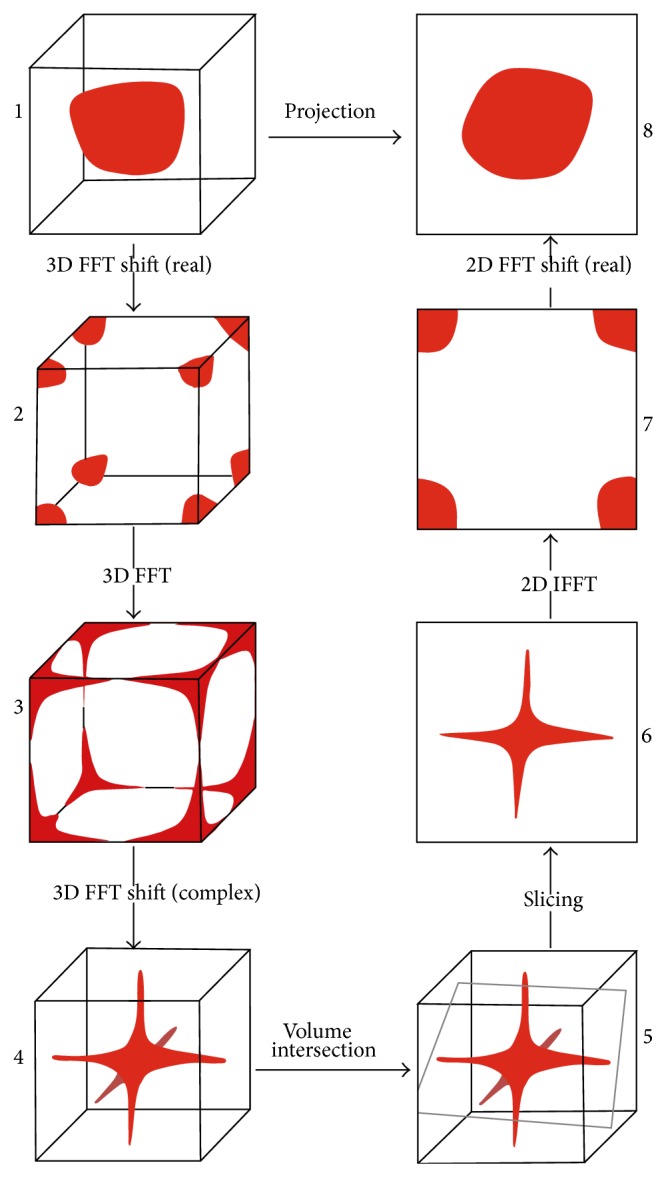
Graphical illustration of the FVR algorithm.

The spatial volume must be shifted by a 3D* fft-shift* operation to set its center at the origin of the 3D space. The frequency spectrum of this volume is then obtained by a forward 3D FFT operation. This resulting spectrum is not centered at the origin of the frequency space. Consequently, another 3D fft-shift operation is required to center its zero-frequency component to prepare it for correct slice extraction. Afterwards, a 2D projection-slice passing through the origin of the spectral volume, with a normal that is parallel to the viewing direction, is carefully extracted. This slice represents the 2D FFT of the desired projection and thus, the reconstructed image can be directly obtained by a 2D inverse FFT operation. To reduce the aliasing and ghosting artifacts in the reconstructed image, this projection-slice is processed in a further step to have it resampled. The resampling stage is only mandatory if the desired projection was not orthogonal. After resampling the extracted slice, it is backtransformed to the spatial domain by an inverse 2D FFT operation. The resulting image from this inverse transformation is shifted by another 2D fft-shift operation to move the center of the image from the edge to origin of the grid used to display the image. Changing the viewing angle implies rotating the 3D spectrum to extract a new projection-slice that corresponds to this angle. The algorithm sequence is graphically depicted in Figure 1.

### 3.2. Pipeline Classification

According to the sequence of operations in this algorithm, the pipeline could be divided into two consecutive stages: a* preprocessing* stage and a* rendering loop*. The preprocessing stage is executed only once to prepare the 3D spectrum of the input spatial volume. The rendering loop is running continuously to generate different projections according to the input viewing angle. The main function of the preprocessing stage is limited to loading a volume of interest, obtaining its frequency spectrum and preparing it for slicing. After the preparation of the spectral volume, the rendering loop is executed to generate different projection images by extracting a projection-slice according to the input viewing angle, then resampling it, and finally backtransforming it to the spatial domain to generate the reconstructed image.

From another perspective, the pipeline could be split according to functionality into two complementary cores or contexts: a* computational* context and a* rendering* context. Cooperatively, both contexts complement each other functional-wise, but from the sequence point of view, they are overlapping and one must switch from one context to the other to flow through the pipeline.

### 3.3. Implementation Strategy

Building the entire FVR pipeline on the GPU directly may be cumbersome and inefficient. In general, an adequate way of constructing GPU-based pipelines is to have a naïve and valid reference implementation of this pipeline on the CPU in advance. Having this CPU-based pipeline tested and validated, every stage of it can be afterwards implemented independently on the GPU and then validated to ensure similar results from the CPU-based implementation until getting the pipeline working integrally on the GPU. This implementation strategy is a rule of thumb to increases flexibility and efficiency for designing and building GPU pipelines. Our proposed FVR pipeline was built based on this strategy.

According to the aforementioned contextual functional classification of the FVR pipeline, the CPU is employed for executing the computational context while the GPU can be used to implement the rendering contexts relying on any graphics application programming interface (API) like OpenGL or Direct 3D. This heterogeneous implementation is easier to handle than a full CPU-based pipeline because the 3D spectral volume can be efficiently represented by 3D OpenGL textures. This efficiency comes from their hardware-acceleration support and built-in interpolation schemes compared to using a 3D array on the CPU and doing the interpolation step manually.

### 3.4. Hybrid Pipeline

**Figure 2:**
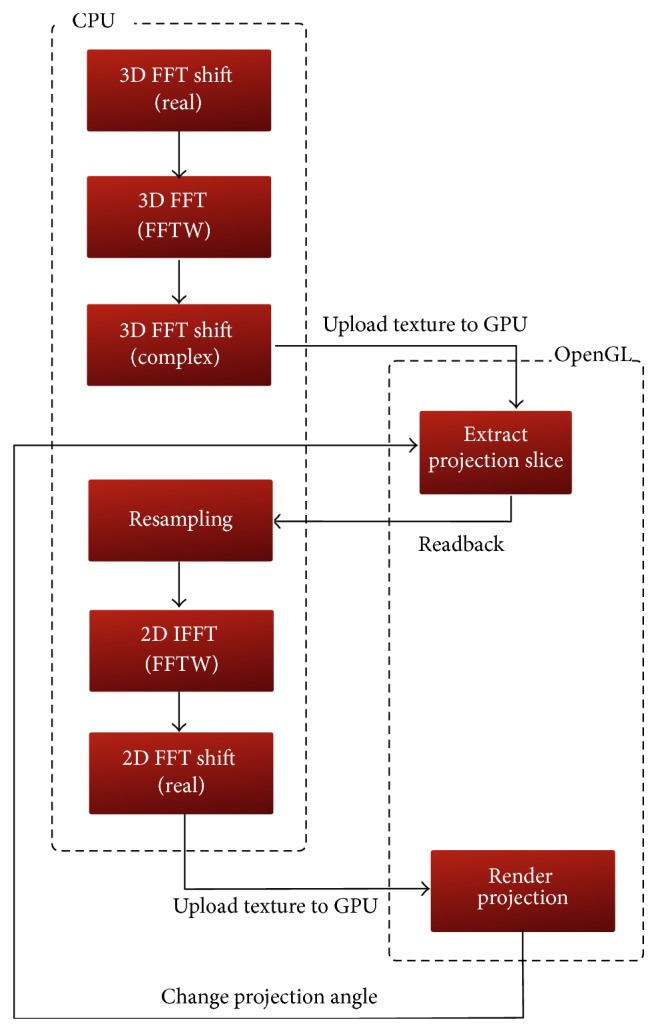
Heterogeneous implementation of the FVR pipeline. The computational context is executed on the CPU and the rendering contexts are executed in OpenGL contexts on the GPU.

**Figure 3:**
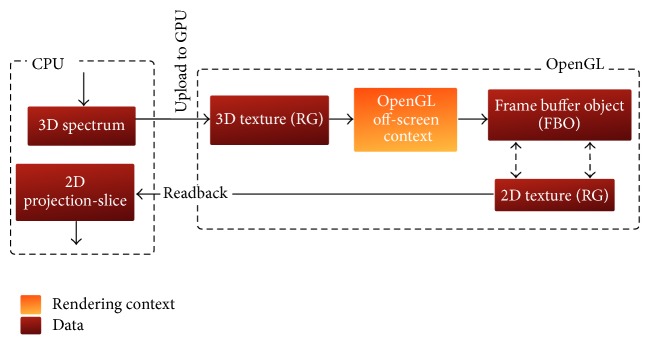
Communication between the computational context and OpenGL off-screen rendering context.

The reference hybrid implementation of the FVR pipeline has been adopted from a previous single threaded one discussed in [24]. This implementation is graphically illustrated in Figure 2. It starts its computational context on the CPU by loading a 3D datasets. This volume is then spatially shifted and its frequency spectrum is obtained by a forward 3D FFT operation. After activating the first OpenGL off-screen rendering context, this spectral volume is mapped to a 3D texture that is allocated on and uploaded to the GPU memory. This texture is intersected with a proxy quadrant passing through the origin of the frequency domain. The resulting projection-slice is then directed to a frame buffer object (FBO) and packed in a 2-component 2D texture attached to this FBO. The contexts are then switched to move back to the computational context on the CPU, where the extracted projection-slice is downloaded from the 2D texture. The communication between the computational and off-screen rendering contexts is shown in Figure 3.

**Figure 4:**
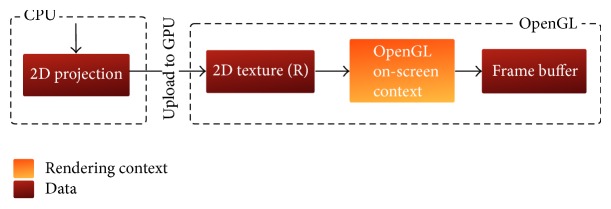
Communication between the computational context and OpenGL on-screen context.

Afterwards, the computational context is resumed by resampling the projection-slice to remove the ghosting artifacts. The resampled slice is then back-transformed by an inverse 2D FFT operation to the spatial domain to produce a shifted image of the projection. This image is rearranged by a 2D fft-shift operation. The final reconstructed image is then packed into a 2D OpenGL texture and uploaded via the command* glTex2D* to reside on the GPU memory. Finally, the on-screen rendering context is activated and the texture that contains the final projection image is sent to the frame buffer to be displayed. This context is illustrated in Figure 4.

### 3.5. Hybrid Implementation Bottlenecks

As usual, each naïve approach has its accompanied bottlenecks that have to be investigated for either their complete removal or at least minimizing their performance overheads. The hybrid implementation of this technique obviously lacked the soul of interactivity due to several bottlenecks. In this section, the hybrid pipeline is analyzed to suppress its main bottlenecks and also to optimize its flow in order to get ready to have it entirely mapped to the GPU with both of its computational and rendering contexts.

A main concern that significantly affects the performance in the computational context is the FFT operations. These operations were expressed relying on the FFTW library [25–29]. The 3D FFT operation takes a considerable amount of time if the size of the input volume was 64^3^ or more. Although it is executed once during the pre-processing stage, performing this operation on several volumes to reveal any depth cues will introduce a real bottleneck and this will consequently elongate the pre-processing stage. Additionally, the inverse 2D FFT operation is executed on a per-frame basis. In turn, reducing the time consumed by this operation will be reflected as an order of magnitude enhancement in the frame rate.

The performance of the rendering loop is affected by the resampling stage, which represents the most critical bottleneck in this pipeline. This operation involves four nested* for* loops. Two of them are used to iterate on each dimension of the slice and the other two are used for the filter kernel. For a projection-slice of size 512^2^, the resampling operation takes a considerable amount of time that eventually blocks a real-time rendering loop. Additionally, executing the computational context on the CPU and the rendering ones on the GPU requires communicating each other to transfer the data back and forth between them. This transfer limits the overall performance of the pipeline to the bandwidth of the communication channel between the CPU and the GPU, which is much less than that on the GPU internally.

### 3.6. Algorithm Mapping to the GPU

Mapping the FVR pipeline to be entirely running on the GPU is a significant step that will leverage the overall performance of the reconstruction process. This mapping procedure is feasible, yet, challenging. Although programming the GPU for executing nongraphics algorithms has been simplified by the introduction of generic GPU APIs like CUDA and OpenCL, but it is far from trivial to effectively exploit this titanic computing power provided by the GPU [30].

The hybrid implementation had a similar flow of typical OpenGL applications. This point makes the communication between OpenGL contexts and the CPU comparatively simple. In the GPU-based approach, the implementation of OpenGL contexts remains the same as that of the previous one, while the computational core of the pipeline will be implemented in a CUDA context. However, both contexts run on the GPU, but the interoperability between them takes a different mechanism and some workarounds for successful and efficient communication. The issues behind OpenGL interoperability with CUDA are discussed with some sort of details in [31, 32].

### 3.7. CUDA Kernels

The first stage for mapping the computational core of the pipeline into a CUDA context is writing analogous device kernels to the respective C functions of the CPU-based computational context. In our mapping, the following kernels have been written.

1. FFT_SHIFT_3D_REAL, which wraps around the 3D spatial volume.
2. FFT_SHIFT_3D_COMPLEX, which wraps around the 3D complex spectrum.
3. FFT_SHIFT_2D_REAL, which wraps around the resulting image from the inverse 2D FFT operation.
4. RESAMPLE_SLICE, which executes the high-order resampling operation.
5. REPACK_ARRAY, which replaces the complex array resulting from the 3D CUFFT operation by another 1D alternative to match the format of the OpenGL 3D spectrum texture.

The fft-shift kernels have been adopted from the proposed implementations in [33, 34]. The following FFT kernels were designed to encapsulate the FFT plans that were originally implemented within the CUFFT library [35].

1. CUDA_FFT_3D, which executes the forward 3D FFT operation.
2. CUDA_FFT_2D, which executes the inverse 2D FFT operation.

### 3.8. GPU-Based Pipeline

**Figure 5:**
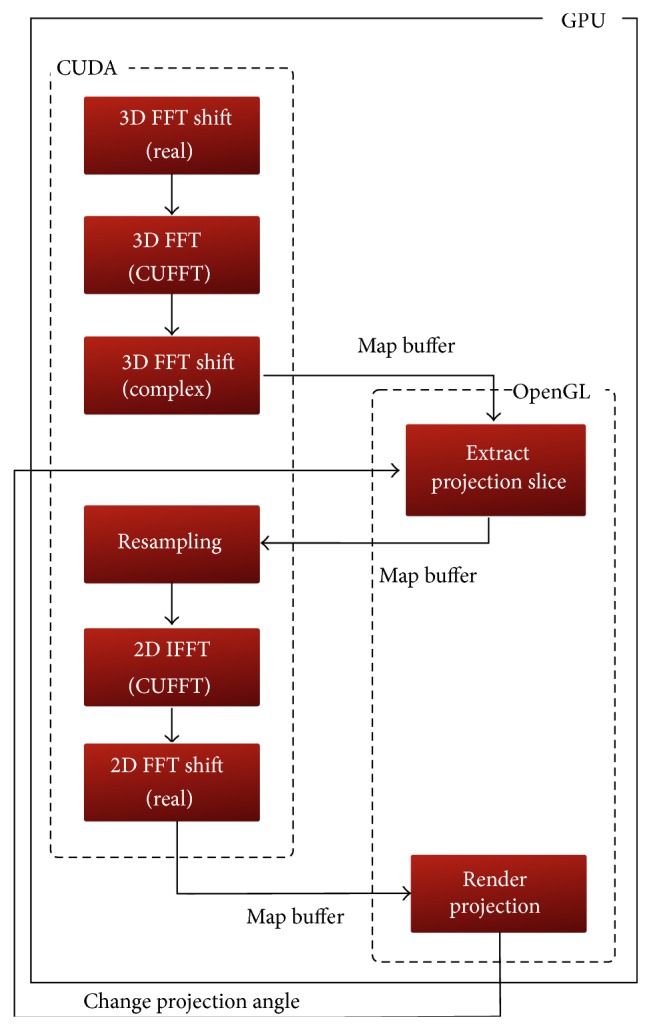
GPU-based implementation of the FVR pipeline. The rendering contexts of the hybrid implementation are reused and the computational one is reimplemented in a CUDA context.

In this GPU-accelerated pipeline, shown in Figure 5, the CPU is only used for loading the input volume and controlling the flow of the pipeline. This control includes switching between the different contexts, copying data from the CPU to the GPU, and dispatching OpenGL commands. Nevertheless, the entire flow of the pipeline is started and terminated on the GPU. The OpenGL rendering contexts employed in the hybrid pipeline will be reused in this implementation to interoperate with the CUDA computational context. However, there will be slight modifications concerned with the communication mechanisms between the different contexts.

Once the volume dataset of interest is loaded to the CPU side, it is directly shoved to reside on the GPU global memory by a* cudaMemcpy* operation. On the GPU side, a CUDA context is created and activated. Then the FFT_SHIFT_3D_REAL kernel is executed for centering the 3D spatial volume. CUDA_FFT_3D function is then invoked to obtain the 3D frequency spectrum of the input spatial volume. Afterwards, the FFT_SHIFT_3D_COMPLEX kernel is invoked to set the center of the spectral volume at the origin of the* k*-space. This array will be mapped to the OpenGL off-screen rendering context for performing the projection-slice extraction operation.

**Figure 6:**
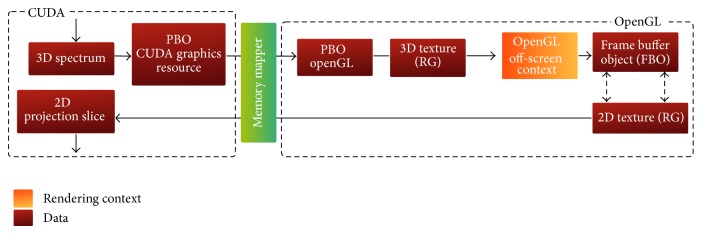
Communication between CUDA and OpenGL off-screen rendering contexts. The spectral volume is mapped between the two contexts via PBOs and the projection-slice is mapped to a 2D texture via CUDA array.

This complex array is converted to an alternative one that is compatible with an OpenGL 3D texture with two components using the REPACK_ARRAY kernel. Although the origin-aligned spectral volume array is located on the GPU memory, it cannot be accessed directly by OpenGL because it is located in the CUDA address space. This array can be read back to the CPU and then uploaded again to the GPU to reside on the texture memory of the CUDA context. This memory transfer operation is extremely useless and can be solved by using an OpenGL pixel buffer object (PBO) that is registered in the CUDA memory space. This workaround makes it possible to directly copy the spectral array to a buffer that is shared between the different contexts of the pipeline. This mechanism is illustrated in Figure 6.

After mapping the spectral texture to OpenGL memory space, we have to switch contexts to load the OpenGL off-screen rendering context. The 3D spectral texture is bound and intersected with a polygon, with the same sizes of the reconstructed image, and the result of this intersection is directed to the FBO and stored in the attached 2D texture. By projecting the desired slice in this FBO, the OpenGL off-screen rendering context is terminated and the rendering pipeline switches back to the CUDA context. A reference to the attached texture to the FBO is mapped to the CUDA context using CUDA array in order to be accessible for subsequent kernel calls. This CUDA array that carries the projection-slice is then mapped to a CUDA texture object. The RESAMPLE_SLICE kernel is then invoked, so the resampling operation is performed on this texture object and the results are stored directly into another 1D array with compatible format with the next CUFFT operation. Afterwards, the CUDA_FFT_2D function is executed on the resampled slice to generate the projection image. This resulting image is shifted, and thus, an fft-shift operation is considered by invoking the FFT_SHIFT_2D_REAL kernel. This correct image is ready to be displayed, but it has to be mapped in advance to an OpenGL 2D texture. This requires mapping the resulting array from the previous fft-shift operation to an OpenGL texture using a PBO.

**Figure 7:**
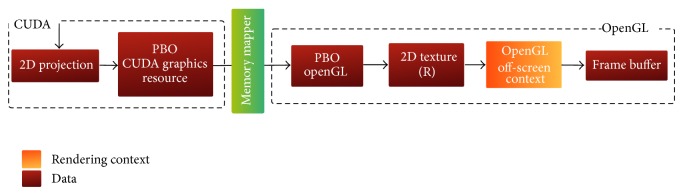
Communication between CUDA and OpenGL on-screen rendering context. The final projection is mapped between the two contexts via the PBO.

After this mapping operation, the on-scree OpenGL rendering context is activated and the final image is pushed to the frame buffer for display. This context is illustrated in Figure 7. Changing the viewing angle repeats this flow by switching back to the OpenGL off-screen context to extract another projection-slice going through all the subsequent stages over and over until the termination of the running process.

### 3.9. GPU-Based Implementation Considerations

Although the OpenGL off-screen context is implemented the same way as it was done in the hybrid pipeline, the extracted projection-slice in the frame buffer object can not be read back by the CPU any more. Alternatively, it is mapped directly to the CUDA memory space by creating another PBO and registering this buffer object with the CUDA memory space. This allows direct attachment of the extracted slice data from the OpenGL off-screen context to be accessible and callable from the different CUDA kernels.

Due to the optimization of the texture caches for 2D spatial locality, reading device memory via texture fetches is more performing than reading from the global memory [36]. In that essence and to exploit the low-latency memory access associated with the texture memory, the extracted projection-slice is directly packed in a CUDA array. This array can be easily mapped to a CUDA texture which is writable from the OpenGL context via the CUDA array. It is only readable from the CUDA side. This texture leads to a significant speed-up in the resampling stage compared to the naïve* for* loop implementation in the hybrid pipeline.

The context switching operations were accelerated due to the direct connection between the different CUDA and OpenGL contexts relying on the shared buffer objects. These buffer objects are registered within all the contexts to allow direct mapping of the resulting textures from the computational context to the rendering ones. This permits efficient data sharing between the different contexts without any memory copies between the CPU and the CPU at all.

## 4. Results and Discussion

### 4.1. Reconstruction Results

**Figure 8:**
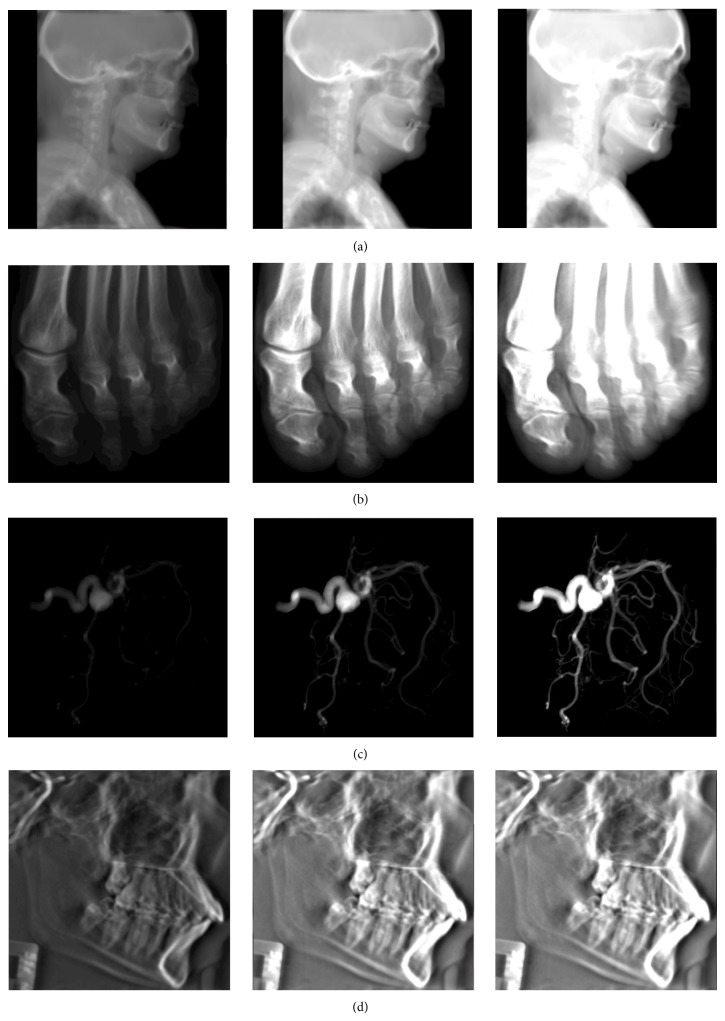
Reconstruction results of rendering several datasets with different projection-slice resolutions and multiple intensity scaling factors. In (a), a sagittal projection of the* visible male* dataset. In (b), an axial projection of a* foot* dataset. In (c), a front view of an* aneurysm* dataset. In (d), a sagittal projection of a* skull* dataset.

Figure 8 shows the resulting radiographs of four medical datasets with different sizes and three intensity scaling factors. All the datasets are organized in regular 3D Cartesian grids [37, 38].

**Figure 9:**
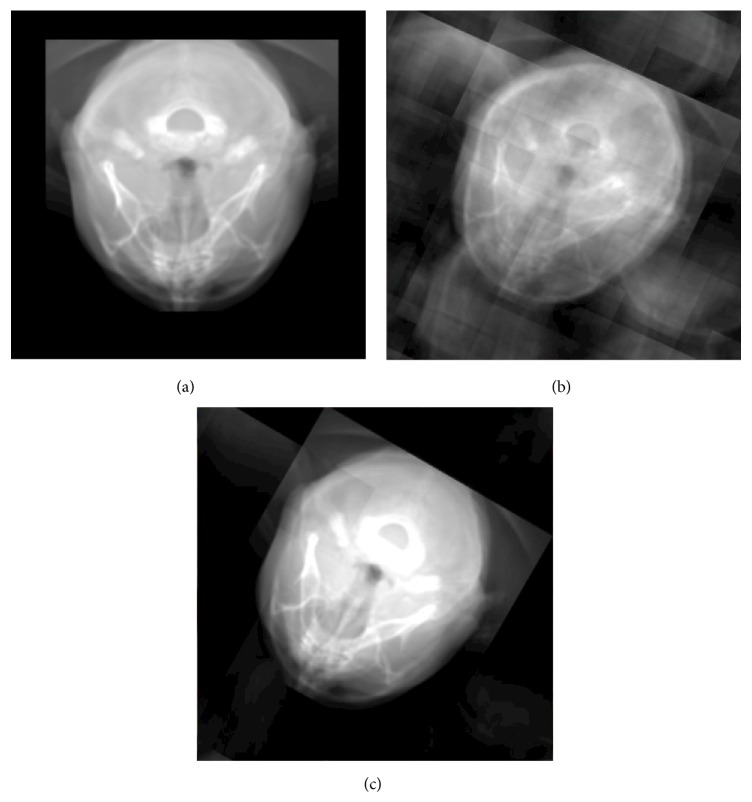
Results of rendering an axial projection of the* visible male* dataset with basic interpolation filters supported by OpenGL 3D textures. In (a), the projection of the dataset is orthogonal and the replicas are not shown. In (b) and (c), the volume is rotated and the ghosting artifacts are very apparent in (b) if a nearest-neighbour interpolation scheme is used and highly reduced if a trilinear interpolation filter is applied as shown in (c). Applying trilinear filtration on the spectral slice significantly affects the rendering performance if the slice size is greater than 512^2^.

To show the artifacts associated with resampling the extracted projection slice, three different interpolation schemes have been applied: point-sampling, trilinear interpolation, and windowed-*sinc* interpolation.  Figure 9 reflects how the selection of the interpolation scheme can significantly affect the rendering quality of the projection image. The projection reconstructed in Figure 9(a) does not exhibit any ghosts because it is orthogonal and all the replicas are not present in the view. In Figure 9(b), the ghosting artifacts are very apparent due to the usage of the point sampling filter, which clamps the missing sample to the nearest available value. In Figure 9(c), the intensities of the replicas are reduced when trilinear interpolation scheme is applied. In fact, the nearest-neighbour and tri-linear interpolation schemes have almost the same performance for resampling projection-slices with sizes less than or equal to 512^2^, but for larger volumes, the performance cost of trilinear interpolation is a bit high. However, this overhead is not significantly degrading the performance of the entire pipeline.

**Figure 10:**
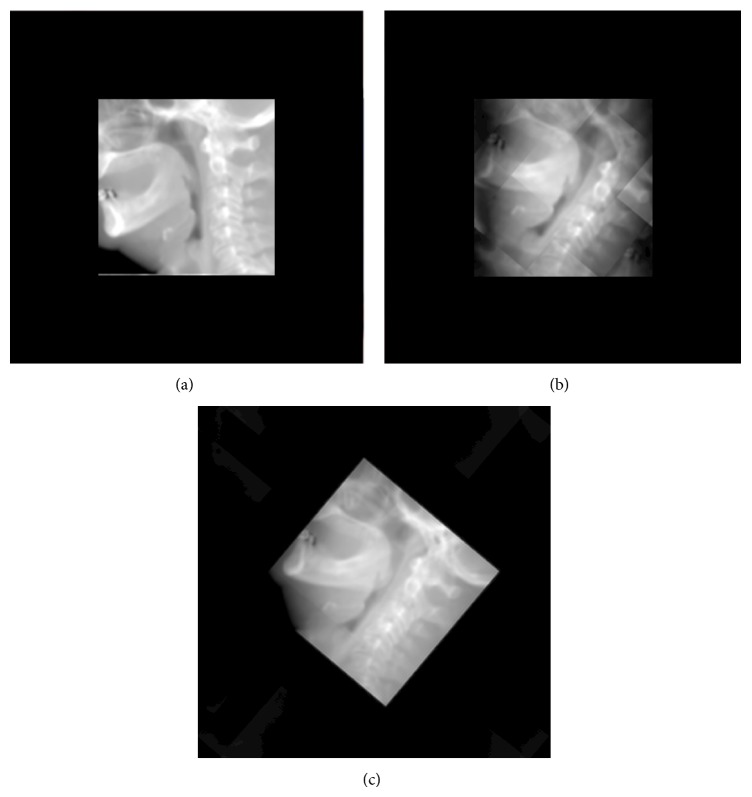
Zero-padding the spatial volume to remove the overlapping between the central image and the replicas. In (a), the central part of the* visible male* dataset is packed in a volume array of the same size. Rotating the volume in (b) results in overlapping. Zero-padding the subvolume removes all the overlapping after rotating the whole volume as shown in (c).

Figure 10 shows the result of zero-padding the spatial volume to suppress the overlapping between the central projection image and the surrounding replicas in the spatial domain. It has to be noticed that the zero-padding operation comes with no overhead on the performance although it dramatically increases the memory requirements by a factor of 8 for 100% zero-padding.

**Figure 11:**
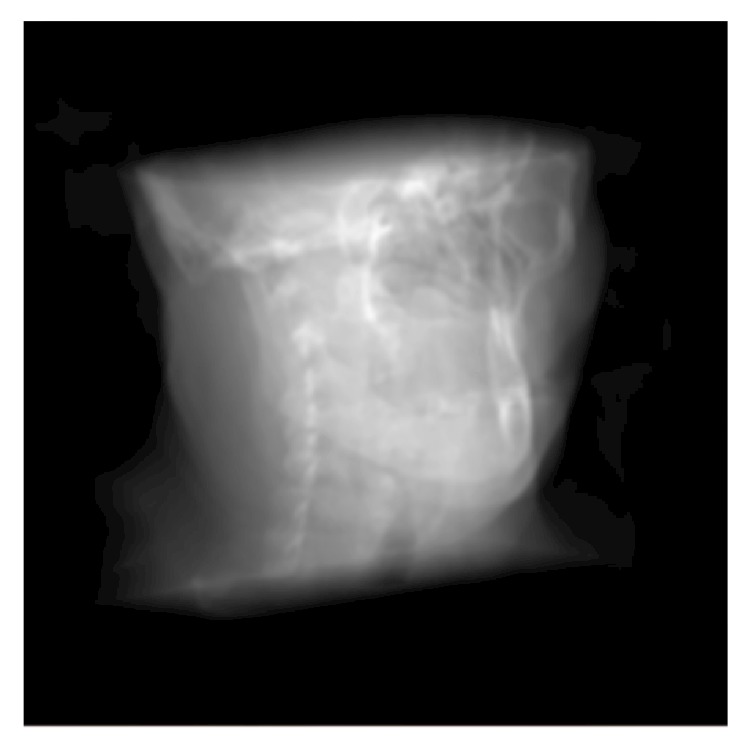
Rendering an oblique projection of the central part of the* visible male* dataset. The volume is zero-padded and the extracted slice undergoes a high-order resampling by applying a Hamming windowed-*sinc* reconstruction filter with order 5 to maximally reduce the ghosting artifacts.

To minimize the artifacts associated with the nature of the technique, the rendering pipeline should afford a high order interpolation filter combined with a preprocessing zero-padding operation. A practical filter that can perform this operation is a Hamming windowed-*sinc* interpolation filter with width 5.  Figure 11 shows the result of combining this reconstruction filter with zero-padding the spatial volume on an oblique projection of the* visible male* datasets.

### 4.2. Performance Analysis

On the performance side and in order to highlight the improvements gained by porting the computational context of the pipeline from the CPU to an alternative CUDA context, we have analysed the profiling results for every stage individually and then we demonstrate and accounted for the overall speed-ups gained for the entire pipeline.

The rendering pipeline has been benchmarked on a workstation shipped with an Intel Core i7-4770 CPU running at 3.4 GHz with 8 MByte of cache and 12 GBytes of DDR3 memory. The application was compiled on Ubuntu 14.04 using GCC 4.7.3 and NVIDIA CUDA compiler nvcc 6.0.1. Two GPUs have been selected to profile the accelerated pipeline. The first one was an NVIDIA GeForce GT 640, which is considered a midrange commodity GPU that costs now almost $100. This GPU has 384 CUDA cores and four GBytes of 128-Bit DDR3 memory. It has been referred to in the text by GPU 1. The other GPU was an NVIDIA QUADRO K5000. This one is much more powerful than the first GPU and can be ranked as a state-of-the-art one since it has 1536 CUDA cores and four GBytes of GDDR5 memory. It will be referred later in the text and the benchmarks by GPU 2.

**Figure 12:**
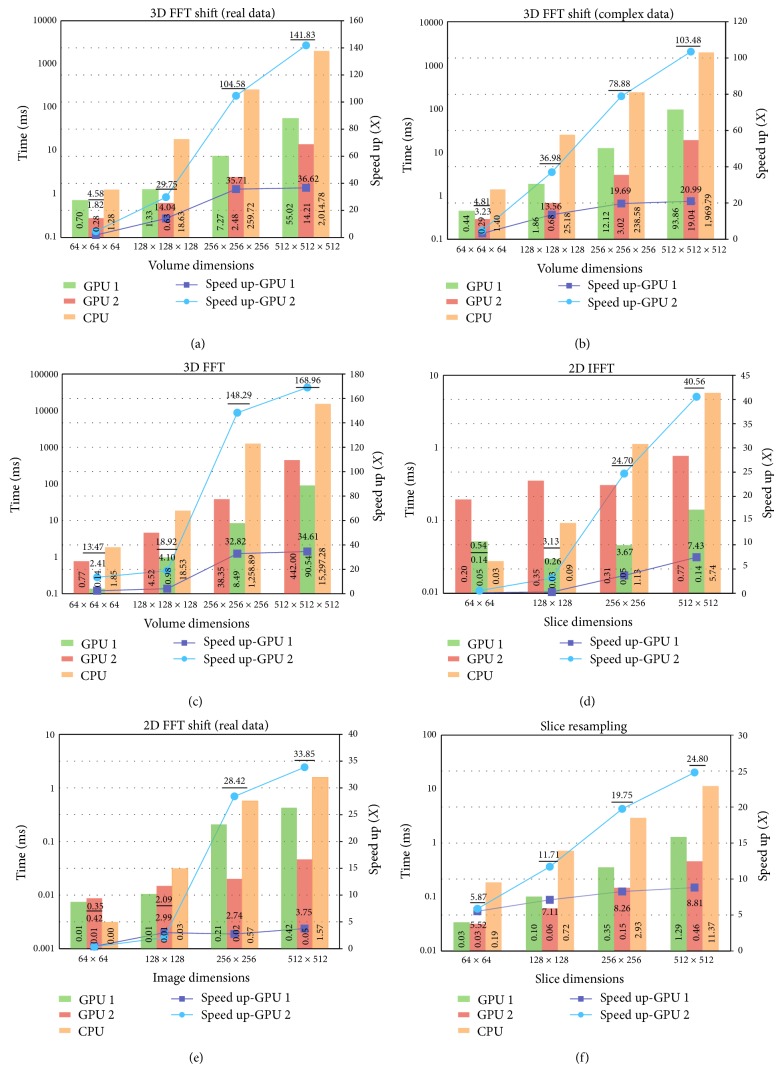
Benchmarking results of the different stages of the rendering pipeline executed on two different GPUs. GPU 1 is an NVIDIA GeForce GT 640, and GPU 2 is an NVIDIA QUADRO K5000.

Additionally, and on the software side, the application was tested with CUDA 6.0. The benchmarks were generated for different volumes of sizes 128^3^, 256^3^, and 512^3^. Although higher speed-ups can be achieved if the volume size goes beyond this limit, unfortunately, larger volumes cannot fit in the memory of any of the employed GPUs.  Figure 12 shows the benchmarking results for every stage in the pipeline to elaborate the difference between the CPU performance in comparison to the two GPUs.

Table 1 summarizes the average execution times for the different stages of the pipeline and aggregates the entire pipeline performance for a volume dataset of size 512^3^. We also reference the hybrid implementation to relatively show that our implementation has gained a speed-up of 117x for the entire pipeline and 29x for the rendering loop. The GPU-accelerated pipeline requires uploading the spatial volume to the device global memory. This step takes on average less than 100 and 40 milli-seconds for a volume of size 512^3^ on GPU 1 and GPU 2, respectively. Thanks to the interoperability mechanisms between CUDA and OpenGL, there are no further data transfer operations between the different stages of the rendering pipeline.

These profiles can be reduced if compared to a multithreaded implementation. However, building a multithreaded OpenGL application is quite complex, and thus a pure GPU implementation is much more appropriate to consider rather than constructing a multithreaded application for benchmarks comparison.

## 5. Conclusion and Future Work

This paper presented a high performance implementation of the Fourier volume rendering algorithm on CUDA-capable GPUs. The literature of FVR was briefly covered and followed by a detailed explanation of the plain rendering algorithm by introducing the notions of computational and rendering contexts. Our implementation strategy considered in advance building a reference hybrid rendering solution where the computational context is executed on the CPU and the rendering one on the GPU using OpenGL. This reference pipeline was then imported in a step-wise fashion to run entirely on the GPU by mapping the computational context to run within a CUDA context and redesigning the rendering contexts to communicate between CUDA and OpenGL to optimize the performance of the rendering loop. Using a 512^3^ dataset, the pure GPU implementation outperformed the hybrid one by a factor of 117x for the entire pipeline and nearly 29x of speed up for the rendering loop. The GPU-accelerated pipeline will be extended in the future to include depth cues and different shading models that can improve the visual appearance of the resulting digital radiographs. A client-server distributed model of the pipeline will be also considered to allow rendering large-scale datasets on multiple computing nodes.

## Figures and Tables

**Table 1:** Performance benchmarks comparison between the different stages of the computational context on the CPU versus GPU for processing a volume of 512^3^. All the results are in *milli-seconds*.

Operation	CPU	GPU 1	GPU 2
Volume uploading to GPU	—	100	40
3D real FFT shift	2014.778	55.01	14.2
3D FFT	15297.28	442.004	90.53
3D complex FFT shift	1969.79	93.86	19.03
Resampling	11.3	1.290	0.458
2D FFT	5.736	0.771	0.1414
2D real FFT shift	1.565	0.4175	0.04623
Preprocessing stage	**19281.847**	**690.886**	**163.78**
Rendering loop	**18.672**	**2.4796**	**0.6462**
Entire pipeline	**19300.5194**	**693.365**	**164.4**

## Acronyms

1D: One-dimensional
2D: Two-dimensional
3D: Three-dimensional
API: Application programming interface
CPU: Central processing unit
CUDA: Compute unified device architecture
DRR: Digital reconstructed radiograph
FDVR: Frequency domain volume rendering
FFT: Fast Fourier transform
FBO: Frame buffer object
FVR: Fourier volume rendering
GPU: Graphics processing unit
PBO: Pixel buffer object.

## References

[1] Elvins T. T. (1992). A survey of algorithms for volume visualization. *ACM SIGGRAPH Computer Graphics*.

[2] Ikits M., Kniss J., Lefohn A., Hansen C. (2007). Volume rendering techniques. *GPU Gems*.

[3] Preim B., Bartz D. (2007). *Visualization in Medicine: Theory, Algorithms, and Applications*.

[4] Dougherty G. (2009). *Digital Image Processing for Medical Applications*.

[5] Kim K. H., Kwon M. J., Kwon S. M., Ra J. B., Park H. W. (2002). Fast surface and volume rendering based on shear-warp factorization for a surgical simulator. *Computer Aided Surgery*.

[6] Udupa J. K. Surface versus volume rendering: a comparative assessment.

[7] Hansen C. D., Johnson C. R. (2005). *The Visualization Handbook*.

[8] Dunne S., Napel S., Rutt B. (1992). Interactive display of volumetric data by fast fourier projection. *Computerized Medical Imaging and Graphics*.

[9] Malzbender T. (2000). Fourier volume rendering. *ACM Transactions on Graphics*.

[10] Grosso R., Ertl T. (1995). Biorthogonal wavelet filters for frequency domain volume rendering. *Visualization in Scientific Computing ’95: Proceedings of the Eurographics Workshop in Chia, Italy, May 3–5, 1995*.

[11] Theußl T. (1999). An implementation of frequency domain volume rendering using the Hartley transform. *The Course of Special Topics in Computer Graphics*.

[12] Levoy M. Volume rendering using the Fourier projection-slice theorem.

[13] Totsuka T., Levoy M. Frequency domain volume rendering.

[14] Entezari A., Scoggins R., Moller T., Machiraju R. Shading for Fourier volume rendering.

[15] Scoggins R. K., Machiraju R., Moorhead R. J. Approximate shading for the re-illumination of synthetic images.

[16] Nagy Z., Novotni M., Klein R. Enhancing Fourier volume rendering using contour extraction.

[17] Cheng C.-C., Ching Y.-T. (2011). Real-time adjustment of transfer function for Fourier volume rendering. *Journal of Electronic Imaging*.

[18] Jansen T., von Rymon-Lipinski B., Hanssen N., Keeve E. Fourier volume rendering on the GPU using a split-stream-FFT.

[19] Ntasis E., Maniatis T. A., Nikita K. S. (2002). Fourier volume rendering for real time preview of digital reconstructed radiographs: a web-based implementation. *Computerized Medical Imaging and Graphics*.

[20] Corrigan A., Wallin J. Visualization of meshless simulations using Fourier volume rendering.

[21] Corrigan A., Wallin J., Vesenjak M. (2009). Visualization of meshless simulations using Fourier volume rendering. *Progress on Meshless Methods*.

[22] Abdellah M., Eldieb A., Sharawi A. Matlab-based Fourier volume rendering framework.

[23] Viola I., Kanitsar A., Gröller M. E. GPU-based frequency domain volume rendering.

[24] Abdellah M., Eldeib A., Sharawi A. Offline large scale fourier volume rendering on low-end hardware.

[25] Frigo M., Johnson S. G. FFTW. http://www.fftw.org/.

[26] Frigo M., Johnson S. G. (1997). The fastest Fourier transform in the west. *Technical Report*.

[27] Frigo M., Johnson S. G. FFTW: an adaptive software architecture for the FFT.

[28] Johnson S. G., Frigo M., Burrus C. S. (2008). Implementing FFTs in practice. *Fast Fourier Transforms*.

[29] Frigo M., Johnson S. G. (2005). The design and implementation of FFTW3. *Proceedings of the IEEE*.

[30] Kirk D. B., Hwu W. W. (2010). *Programming Massively Parallel Processors: A Hands-on Approach*.

[31] Stam J. http://developer.download.nvidia.com/compute/cuda/docs/GTC09Materials.htm.

[32] Farber R. (2011). *CUDA Application Design and Development*.

[33] Abdellah M., Saleh S., Eldeib A., Shaarawi A. High performance multi-dimensional (2D/3D) FFT-Shift implementation on Graphics Processing Units (GPUs).

[34] Abdellah M. cufftShift: high performance CUDA-accelerated FFT-shift library.

[35] NVIDIA CUFFT library.

[36] NVIDIA (2012). *CUDA C Best Practice Guide (Design Guide)*.

[37] The Volume Library Online library for Volume visualization datasets. http://lgdv.cs.fau.de/External/vollib/.

[38] Online library for medical MR and CT datasets. http://www.volvis.org/.

